# Nivolumab-related myasthenia gravis with myositis requiring prolonged mechanical ventilation: a case report

**DOI:** 10.1186/s13256-022-03286-x

**Published:** 2022-02-14

**Authors:** Yumi Saishu, Takuya Yoshida, Yusuke Seino, Takeshi Nomura

**Affiliations:** Department of Intensive Care Medicine, Tokyo Women’s Medical University, 8-1 Kawada-cho, Shinjuku-ku, Tokyo, 162-8666 Japan

**Keywords:** Nivolumab, Myasthenia gravis, Melanoma, Diaphragm ultrasound

## Abstract

**Background:**

Nivolumab is an immune checkpoint inhibitor that blocks inhibitors of T-cell activation and blunts antitumor immunity and is used in the treatment of various cancers. However, immune checkpoint inhibitors have immune-related adverse effects on various organs due to promoting T-cell activity against host tissues by blocking inhibition of T-cell function. Although immune-related adverse effects including hepatitis, colitis, pneumonitis, dermatitis, nephritis, endocrinopathies, and hypophysitis are well recognized with established treatment guidelines, neuromuscular immune-related adverse effects are rare phenomena.

**Case presentation:**

A 55-year-old Asian (Japanese) woman was diagnosed with nivolumab-related myasthenia gravis with myositis and myocarditis. She had a past history of thymectomy for large thymoma with a high anti-acetylcholine receptor antibody level without any symptoms. Nivolumab was administered for the treatment of malignant melanoma. Creatine kinase levels began to rise 2 weeks after the administration, and abnormal neurological findings appeared 3 weeks after the administration. Ventricular arrhythmia, wide QRS complex, and dyssynchrony of the left ventricle also appeared. Intravenous immunoglobulin and corticosteroids were administered, and plasma exchange was performed. The patient required intensive care and prolonged mechanical ventilation with tracheostomy owing to weakness of the diaphragm; she was eventually weaned from the ventilator and discharged. Diaphragm ultrasound was used for the decision-making of the weaning strategy and evaluation of the diaphragmatic function.

**Conclusions:**

Nivolumab-induced severe myasthenia gravis with myositis and myocarditis required intensive care and prolonged mechanical ventilation. Although immune checkpoint inhibitor-related myasthenia gravis is a rare adverse event, appropriate and prompt treatment is required because of its severity and rapid progression. Diaphragm ultrasound was useful not only in diagnosing diaphragm dysfunction and deciding the strategy for weaning from mechanical ventilation but also in evaluating the recovery of the diaphragmatic function.

## Background

Nivolumab is an immune checkpoint inhibitor (ICI) that blocks inhibitors of T-cell activation and causes blunting of antitumor immunity [1, 2]. ICIs work against the immune checkpoint modulators such as cytotoxic T-lymphocyte-associated protein 4 (CTLA-4; ipilimumab), programmed cell death protein 1 (PD-1; nivolumab, pembrolizumab), and its ligand (PD-L1; atezolizumab, avelumab, durvalumab), and are used in the treatment of various cancers, including lung cancer, kidney cancer, bladder cancer, prostate cancer, lymphomas, and malignant melanomas [1]. However, ICIs have immune-related adverse effects (iAEs) on various organs due to promoting T-cell activity against host tissues by blocking inhibition of T-cell function [1]. Although iAEs including hepatitis, colitis, pneumonitis, dermatitis, nephritis, endocrinopathies, and hypophysitis are well recognized with established treatment guidelines, neuromuscular iAEs, including myositis, myasthenia gravis (MG), and peripheral neuropathy, are rare phenomena [3, 4].

We present a case of nivolumab-related MG with myositis. The patient required intensive care and prolonged mechanical ventilation (MV) with tracheostomy owing to weakness of the diaphragm; she was eventually weaned from the ventilator and discharged. Diaphragm ultrasound was used for the decision-making of the weaning strategy and evaluation of the diaphragmatic function.

## Case presentation

A 55-year-old Asian (Japanese) woman presented with right lower quadrantanopia. She had a past history of thymectomy for large thymoma and postoperative irradiation 6 years ago and no notable family history. Although the anti-acetylcholine receptor (AChR) antibody level was still high (20 nmol/L) even after surgery, MG had not developed. Head computed tomography (CT) and magnetic resonance imaging (MRI) after the onset of the symptom revealed a tumor in the left parieto-occipital lobe. She underwent craniotomy for tumor resection, and the histopathological findings revealed malignant melanoma. She had had a black nodule on the right chest for the past 6 years, and histopathological examination led to diagnosis of malignant melanoma, which was considered to be the primary tumor. The primary and metastatic brain tumor had already been resected; nevertheless, she and her family wanted to undergo chemotherapy without the target lesion. The first dose of nivolumab (3 mg/kg) was administered (day 1), and the second dose (3 mg/kg) was administered 14 days later (day 15).

Creatine kinase (CK) levels began to rise on day 14 and gradually increased to 6279 IU/L on day 19. At this point, rhabdomyolysis due to nivolumab was suspected because there were no abnormal neurological findings. CK increased to 13,603 IU/L, and right ocular motility disorder, diplopia, and dysphagia appeared on day 21 [quantitative MG (QMG) score 14]. Left ocular motility disorder and muscle weakness of the extremities emerged on day 22. Since myositis and myasthenia gravis due to nivolumab were suspected, intravenous administration of immunoglobulin and prednisolone (20 mg/day) was started even before the definitive diagnosis was made. Bilateral ptosis appeared and muscle weakness developed on days 23 and 24 (QMG score 21 and 24). Multiple premature ventricular contractions, ventricular tachycardia, wide QRS complex in the electrocardiogram, and dyssynchrony of the left ventricle in echocardiography were observed, and myocarditis was also suspected. Based on the findings of positive anti-AChR antibody (29 nmol/L), and inflammation in thigh muscle on MRI, nivolumab-related myasthenia gravis with myositis was diagnosed.

**Fig. 1:**
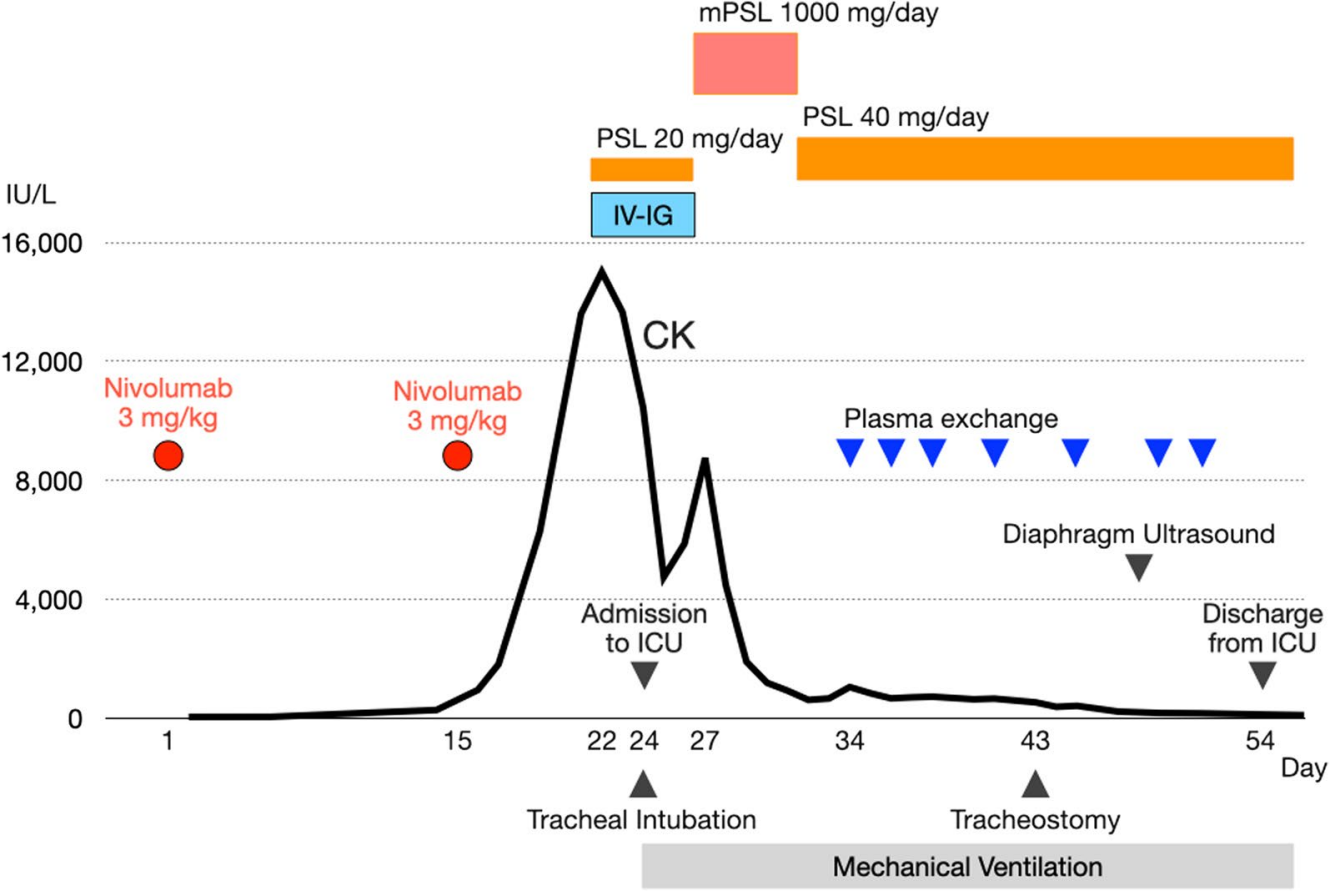
The clinical course and treatment after the administration of nivolumab. *CK* creatine kinase, *ICU* intensive care unit, *IV-IG* intravenous immunoglobulin, *mPSL* methylprednisolone, *PSL* prednisolone

**Fig. 2:**
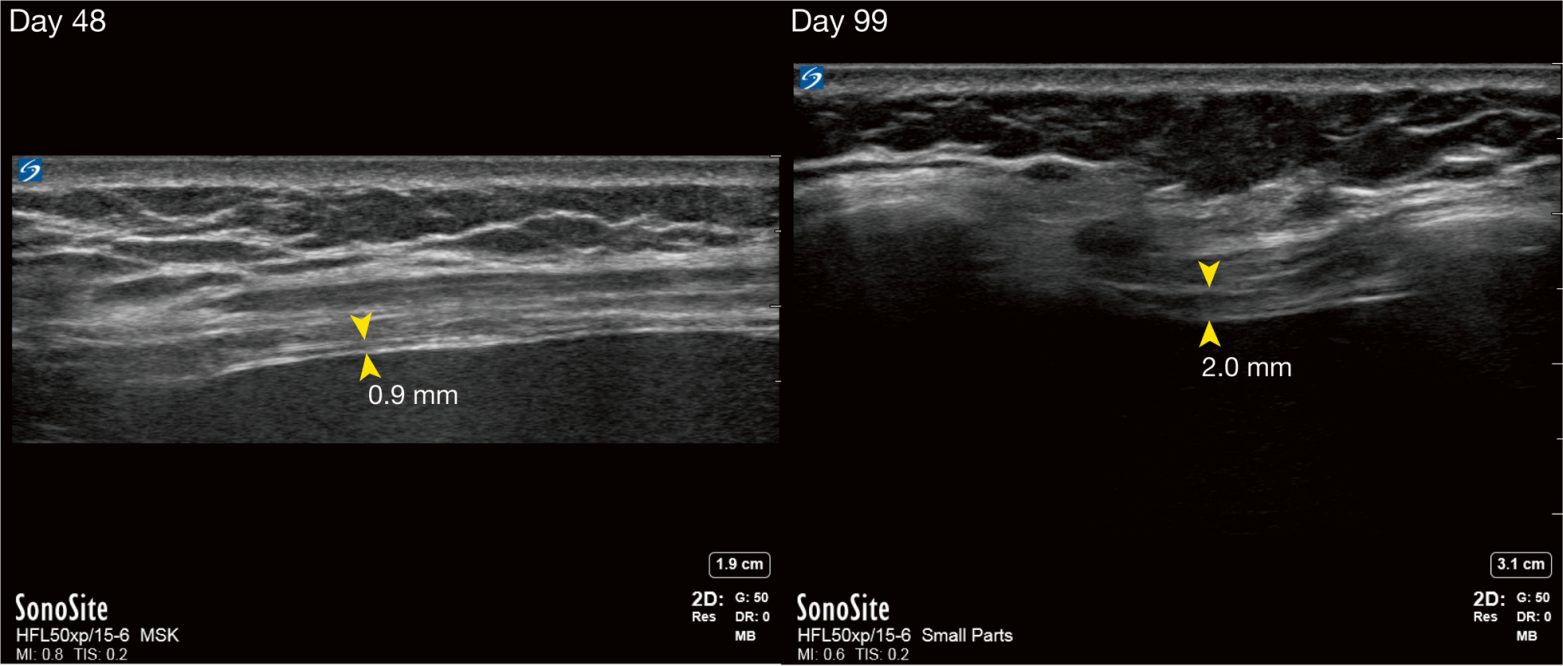
Images of diaphragm ultrasound at the zone of apposition of the right hemidiaphragm on day 48 (left) and day 99 (right). The thicknesses at the end inspiration are shown (arrowheads)

She was admitted to the intensive care unit (ICU) and underwent tracheal intubation for MV on day 24 because of elevated respiratory rate and dyspnea due to respiratory muscle dysfunction. The administration of immunoglobulin and corticosteroids (methylprednisolone and prednisolone) and plasma exchange gradually improved muscle weakness of the extremities, ptosis, ocular motility disorder, and CK elevation (Fig. 1). However, weaning from MV was difficult due to respiratory muscle weakness; thus, tracheostomy was performed on day 43. Diaphragm ultrasound revealed that the diaphragm was extremely thin, and there was no excursion or spontaneous movement (Fig. 2, left). She was discharged from the ICU on MV on day 54.

After discharge from the ICU, intravenous immunoglobulin (5 days, four times) and corticosteroids (methylprednisolone and prednisolone) were administered, and plasma exchange (five times) was performed. The symptoms, including bilateral ptosis, ocular motility disorder, and muscle weakness of the extremities, continuously improved, and she was able to walk with a cane following rehabilitation. There was no finding of recurrence of the symptoms. The level of anti-AChR antibody decreased to 2.9 nmol/L on day 72. However, respiratory muscle function was significantly impaired, and weaning from MV required a long time. The respiratory support team adjusted the ventilator settings for weaning and periodically performed diaphragm ultrasound. The thickness, contraction, and excursion of the diaphragm gradually increased (Fig. 2, right and Table 1). She was able to reduce her ventilatory support and was able to discontinue MV in the daytime. Eventually, she was completely weaned from MV on day 199, and the tracheal cannula was removed on day 216. She was discharged on day 254.

**Table 1:** The findings of diaphragm ultrasound

Day		48	63	77	99	119	146	182
Thickness at end inspiration/end expiration (mm)	Right	0.9/0.9	1.0/1.0	2.3/1.9	2.0/1.3	NA/0.6	1.3/NA	1.5/NA
	Left	0.9/0.9	0.8/0.8	0.8/0.5	1.3/1.3	NA/1.0	1.0/NA	1.8/NA
Thickening	Right	None	None	+	+	NA	+	+
	Left	None	None	None	None	NA	+	+
MV	PS	10	10	10	9	9	7	
	PEEP	5	5	5	5	5	5	

*MV* mechanical ventilation, *NA* not available, *PS* pressure support, *PEEP* positive end-expiratory pressure

## Discussion

We identified two important clinical issues in this case. Nivolumab induced severe MG with myositis requiring intensive care and prolonged MV. Diaphragm ultrasound was useful in diagnosing diaphragm dysfunction, deciding the strategy for weaning from MV, and evaluating the recovery of the diaphragm.

The incidence of severe neurotoxicities of anti-PD-1 and anti-PD-L1 antibodies was very rare (reported to be < 1%) compared with other iAE, including dermatologic toxicities (34–39%), diarrhea/colitis (1–3%), hypophysitis (1–6%), hepatic toxicities (5% or less), and pneumonitis (< 10%) [5, 6]. The neuromuscular iAEs include myositis, MG, and peripheral neuropathies, of which MG is the most frequent manifestation [4, 7]. Safa *et al*. reported that the incidence of ICI-related MG in treated patients was 0.24% (14/5898 patients) at a single center and conducted a systematic review of 65 patients, adding 51 patients reported in the literature [8]. The frequent symptoms included ptosis (75%), dyspnea (62%), limb weakness (55%), dysphagia (48%), and diplopia (42%). Myositis and myocarditis were seen in 24 (37%) and 5 (8%) patients, respectively [8]. Since ICI-related MG (irMG) and ICI-related myositis have overlapping clinical presentations, and irMG has different features from idiopathic MG, there may be a pathological condition where MG and myositis coexist in iAE [9–11]. On the other hand, the study using the 2-year safety database based on postmarketing surveys in Japan reported that the incidence of nivolumab-related MG was 0.12% (12/9869 patients) [10]. In addition, irMG was characterized by acute onset and rapid deterioration compared with idiopathic MG [8, 10].

In the present case, the patient had a history of thymectomy and a high anti-AChR antibody level without MG symptoms. There are some reports of patients with exacerbations of preexisting MG as a result of immune checkpoint inhibition [2]. Suzuki *et al*. reported that there were patients with nivolumab-related MG who had a high anti-AChR antibody level before the onset of MG [10]. Thus, subclinical MG might become apparent by nivolumab administration in such patients rather than completely *de novo* MG directly induced by nivolumab, and careful consideration of the indications of ICIs may be warranted in patients with a history related to MG.

Corticosteroids, intravenous immunoglobulin, acetylcholinesterase inhibitors, plasma exchange, and other immunosuppressants have been used for the treatment of ICI-related MG, adding to the discontinuation of ICI [8, 10]. ICI-related MG sometimes requires intensive care and MV. A review of 65 patients showed that 29 patients (45%) required MV because of respiratory failure, and 10 patients were able to wean from MV [8]. In addition, complete resolution of MG symptoms was seen in 12 patients (19%), improvement in 34 patients (55%), and worsening in 16 patients (26%), and mortality due to MG was 23% (15/65 patients) [8]. The 2-year safety database study in Japan also reported that 5 of 12 patients with ICI-related MG required respiratory support, and 4 patients were able to wean from MV [10]. The study also reported that the mean duration of respiratory support was 54 days, and the patients’ activities were severely impaired for a long duration. Mortality was 17% (2/12 patients) [10]. In the present case, the patient developed severe nivolumab-induced MG with myositis, requiring a tracheostomy and about 200-day MV, but was able to be weaned from the ventilator and was eventually discharged.

Diaphragm ultrasound played a pivotal role in diagnosing dysfunction of the diaphragm, determining the weaning strategy from MV in the ICU, and evaluating the improvement of diaphragm function. There are various methods for evaluating diaphragmatic function, including chest radiography, fluoroscopy, pulmonary function test, pressure-generating capacity, electromyogram, and ultrasound [12, 13]. Diaphragm ultrasound has the advantage of providing serial, noninvasive findings at the bedside of critically ill patients, being a simple procedure with easy learning curve [14]. The excursion, thickness, and thickening of the diaphragm during inspiration were used for evaluating the diaphragmatic function [15]. In the present case, thickening of the diaphragm was mainly used as an indicator of improvement, since the patient was under mechanical support and diaphragmatic excursion depended on the amount of ventilatory support and positive end-expiratory positive pressure [16]. The usefulness of diaphragm ultrasound was reported in patients with difficult weaning to predict successful weaning from the ventilator, and cutoff values have been proposed for excursion thickness and thickening of the diaphragm [17]. In the present case, the thickness and thickening of the diaphragm were so extremely reduced that we gave up on short-term weaning from MV and performed tracheostomy instead.

## Conclusions

Nivolumab-induced severe MG with myositis required intensive care and prolonged MV. Although ICI-related MG with myositis is a rare adverse event, appropriate and prompt treatment is required because of its severity and rapid progression. Diaphragm ultrasound was useful not only in diagnosing diaphragm dysfunction and deciding the strategy for weaning from MV but also in evaluating the recovery of the diaphragmatic function.

## Data Availability

The datasets used and/or analyzed during the current study are available from the corresponding author on reasonable request.

## Abbreviations

AChR: Acetylcholine receptor
CK: Creatine kinase
CT: Computed tomography
CTLA-4: Cytotoxic T-lymphocyte-associated protein 4
iAEs: Immune-related adverse effects
ICI: Immune checkpoint inhibitor
ICU: Intensive care unit
irMG: Immune checkpoint inhibitor-related myasthenia gravis
MG: Myasthenia gravis
MRI: Magnetic resonance imaging
MV: Mechanical ventilation
PD-1: Programmed cell death protein 1
PD-L1: Programmed cell death protein 1 ligand
QMG score: Quantitative myasthenia gravis score
